# Changes in Service Utilization and Unmet Needs in HCBS in the United States During the COVID-19 Pandemic

**DOI:** 10.1093/geroni/igaf122.1311

**Published:** 2025-12-31

**Authors:** Romil Parikh, Tetyana Shippee, Jack Wolf, Eric Jutkowitz, Stephanie Giordano

**Affiliations:** University of Minnesota School of Public Health, Minneapolis, Minnesota, United States; University of Minnesota, Minneapolis, Minnesota, United States; University of Minnesota School of Public Health, Minneapolis, Minnesota, United States; University of Minnesota, Minneapolis, Minnesota, United States; Human Services Research Institute, Cambridge, Massachusetts, United States

## Abstract

More than 5 million older adults in the United States use publicly-funded home-and community-based services (HCBS) which were disrupted during the COVID-19 pandemic. We evaluated changes in HCBS use and consumer-reported unmet HCBS needs during the pandemic (2021-2022) versus pre-pandemic (2018-2019). We evaluated six commonly used HCBS: personal care, homemaker, meal delivery, adult day, transportation, and caregiver respite/support services. We used mixed effects logistic regression modeling to estimate adjusted odds ratios (OR) and 95% confidence intervals (CI), adjusting for consumers’ sociodemographic and health-related characteristics, with random intercepts for state. We included community-dwelling, HCBS consumers (age, ≥65 years) from 11 states that participated in both- 2018-2019 and 2021-2022 survey waves. Compared to 2018-2019, service use increased for personal care (OR, 1.24; 95% CI, 1.09, 1.40) and caregiver respite/support (OR, 1.28; 95% CI, 1.00, 1.63) but decreased for homemaker services (OR, 0.69; 95% CI, 0.60, 0.79) and meal delivery (aOR, 0.81; 95% CI, 0.70, 0.93). In 2021-2022, unmet HCBS needs significantly increased for personal care (OR, 1.23; 95% CI, 1.03, 1.46) and meal delivery (OR, 1.26; 95% CI, 1.01, 1.56) but significantly decreased for caregiver respite/support (OR, 0.49; 95% CI, 0.35, 0.70). During the pandemic, changes in service utilization and consumer-reported unmet service needs differed between various types of HCBS. These findings can inform disaster preparedness efforts for assuring continuity of HCBS during future public health emergencies.

